# Potential of Integrin Inhibitors for Treating Ovarian Cancer: A Literature Review

**DOI:** 10.3390/cancers9070083

**Published:** 2017-07-08

**Authors:** Masaki Kobayashi, Kenjiro Sawada, Tadashi Kimura

**Affiliations:** Department of Obstetrics and Gynecology, Osaka University Graduate School of Medicine, 2-2, Yamadaoka Suita, Osaka 5650871, Japan; macha_111@hotmail.com (M.K.); tadashi@gyne.med.osaka-u.ac.jp (T.K.)

**Keywords:** integrin, ovarian cancer, angiogenesis, RGD peptide, volociximab

## Abstract

Epithelial ovarian cancer is a fatal disease, with a cure rate of only 30%. Several recent studies have targeted integrins for cancer treatment. Preclinical studies have shown the effectiveness of several integrin inhibitors for blocking cancer progression, especially by blocking angiogenesis. Because the initial critical step in ovarian cancer metastasis is the attachment of cancer cells to the peritoneum or omentum and because clinical trials have provided positive results for anti-angiogenic therapy, therapies targeting integrins may be the most feasible approach for treating cancer. This review summarizes the current understanding of integrin biology in ovarian cancer metastasis and various therapeutic approaches involving integrin inhibitors. However, no integrin inhibitor has shown favorable results thus far. However, conjugates of cytotoxic agents with the triplet sequence arginine-glycine-aspartate (RGD) peptides targeting α5β1-, αvβ3-, and αvβ6-integrins may be promising integrin-targeting therapies for further clinical investigation.

## 1. Introduction

Epithelial ovarian cancer (EOC) is one of the most common gynecologic malignancies and the fifth leading cause of cancer-related death in women [1]. EOC is a highly metastatic cancer characterized by widespread peritoneal dissemination and ascites accumulation. Therefore, it is typically diagnosed in advanced stages [2]. The standard treatment of EOC is debulking surgery, followed by repeated courses of platinum- and taxane-based chemotherapy. However, most patients with EOC eventually relapse despite receiving intensive treatment, including several lines of chemotherapy, with a 5-year survival rate of patients with advanced-stage EOC being only 30%. The current treatment of EOC is highly associated with the rapid development of drug resistance. Therefore, new therapeutic strategies, including molecular targeting therapies, have been recently considered for treating EOC [3]. During ovarian cancer dissemination, cells detach from the primary tumor site, which was initially thought to be the ovary but is now believed to be the fallopian tube [4]. Subsequently, ovarian cancer cells float in the peritoneal cavity and attach to secondary sites of implantation, most notably the omentum, which is the most common site of ovarian cancer metastasis [5]. Several integrins have been identified as important mediators of ovarian cancer metastasis to the mesothelium [6], suggesting that use of integrin inhibitors could be a new therapeutic strategy to prevent the attachment of cancer cells to the peritoneal cavity. In this review, we describe the critical roles of integrins in ovarian carcinoma metastasis and recent progress in targeting integrins for treating several cancers, including ovarian cancer.

## 2. Biology of Integrins

**Figure 1:**
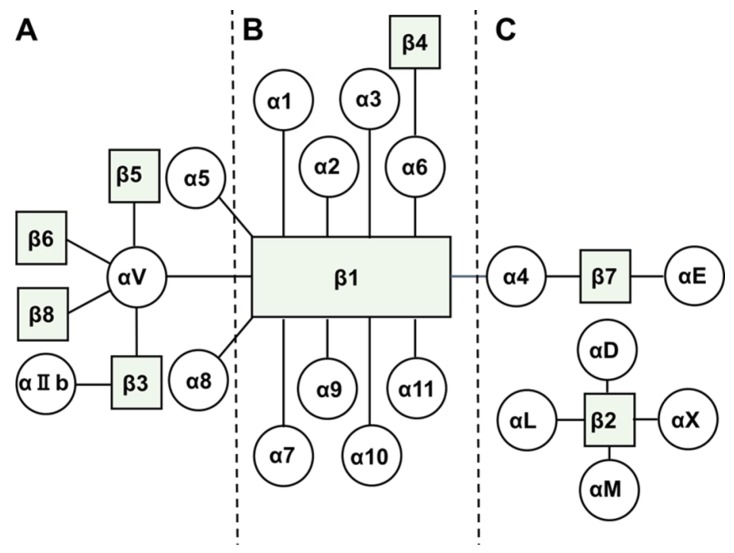
Members of integrin family and their interactions to form heterodimeric integrins [7]. (**A**) Integrins are roughly grouped into three classes and bind to provisional extracellular matrix, (**B**) basal extracellular matrix, and (**C**) cell surface adhesion molecules.

Integrins are heterodimeric adhesion receptors expressed on the cell surface and consist of two non-covalently bound subunits, namely, an α subunit and a β subunit. The α subunit has four extracellular domains, namely, β propeller, thigh, calf-1, and calf-2 domains. The β subunit has an ectodomain containing eight sub-domains, a β1 domain, a hybrid domain, a plexin-semaphorin-integrin domain, a β-tail, and four integrin-epidermal growth factor domains. Thus far, 18 α subunits and 8 β subunits of integrins have been identified, which combine to form 24 different integrin heterodimers with different specificities toward extracellular components such as collagen, laminin, fibronectin, and vitronectin [7] (Figure 1). Many integrins recognize and bind to Arg-Gly-Asp (RGD), Arg-Gly-Asp-Val (REDV) or Glu-Ilo-Leu-Asp-Val (EILDV) motif present in extracellular matrix proteins. For instance, αvβ1, αvβ3, αvβ5, αvβ6, αvβ8, α5β1, α8β1 and αIIbβ3 recognize RGD motif found in fibronectin, collagen, vitronectin, osteopontin and thrombospondin [8]. α4β1-Integrin recognizes REDV and EILDV motif in an alternatively spliced form of fibronectin [9]. Molecular and physical interactions among cancer cells and between cancer cells and the extracellular matrix strongly affect their behavior, thus inducing cancer cell invasion and dissemination [10]. Moreover, cell adhesion is crucial for inflammation, platelet aggregation, and tissue repair. Several previous studies have reported the overexpression of some integrins during cancer development and have targeted overexpressed integrins for treating several cancers, including ovarian cancer [3]. 

## 3. Current Treatment of Ovarian Cancer

EOC is the most common histological type of ovarian cancer, with a 5-year survival of patients with different stages of EOC being approximately 45.6% [11]. EOC is surgically staged based on the International Federation of Gynecologists and Obstetricians criteria, with the 5-year survival rate increasing to >70% in a minority of patients diagnosed at an early stage but decreasing to 35% in majority of patients diagnosed at an advanced stage [11]. In all, 75% patients with ovarian cancers are diagnosed at the advanced stage. EOC is usually insidious, and most patients with EOC show no symptoms until the cancer reaches the advanced stage. Typical symptoms of EOC are pelvic or abdominal pain, frequent urination, constipation, abdominal distension, and early satiety [12].

At present, the first-line treatment for EOC is surgical cytoreduction or removal of grossly evident tumor. The aim of surgery for treating ovarian cancer is optimal surgical cytoreduction, with the reduction of tumor size to <1 cm according to the guidelines of the Gynecologic Oncology Group. Residual disease after surgical cytoreduction is one of the most important prognostic factors of EOC, and aggressive surgical procedures are appropriate for managing this disease [13]. This effect of the surgical cytoreduction is indicative of a dramatic difference in the biological behavior of ovarian cancer as compared with other malignancies, because the removal of metastatic tumors has not been found to improve survival in most other cancers such as colon or gastric cancers. This is because the mechanism underlying the dissemination of ovarian cancer in different from that underlying the dissemination of other malignancies. While most other cancer cells mainly metastasize by using the vasculature, ovarian cancer cells mainly metastasize by directly migrating from the primary tumor site in the ovary or fallopian tube to neighboring organs, including the uterus, bladder, and colon, or by floating in physiological peritoneal fluid. Exfoliated tumor cells from the primary tumor site are transported across the peritoneum and disseminate within the abdominal cavity [14]. After surgical cytoreduction or staging, patients with advanced EOC receive carboplatin-paclitaxel combination chemotherapy. This chemotherapy can be administered as an adjuvant and/or neoadjuvant chemotherapy depending on the successful completion of the cytoreductive surgery [15]. Although most patients with EOC respond to this treatment initially, majority of patients relapse and eventually succumb to the disease [16]. Patients showing resistance to platinum-based chemotherapy have a response rate of <20% toward other agents and show a median survival of approximately 1 year [17].

Because of the dismal cure rate of ovarian cancer resistant to platinum-based chemotherapy, several studies have evaluated the potential of other novel targeting therapies for treating ovarian cancer. Among new drugs assessed for treating ovarian cancer, bevacizumab, a humanized anti-vascular endothelial growth factor (anti-VEGF) monoclonal antibody, has shown promising activity in combination with standard chemotherapy in several phase III trials such as ICON7 and GOG218 trials [18,19]. Bevacizumab blocks new angiogenesis, thus preventing tumor formation by inhibiting the formation of new blood vessels. Several studies have shown that angiogenesis is an important contributor to ovarian carcinogenesis and progression. Of various angiogenic factors, VEGF induces endothelial cell proliferation, promotes cell migration, and inhibits apoptosis; moreover, VEGF is strongly suggested to be a key regulator of physiological and pathological angiogenesis [20]. Therefore, bevacizumab may be a “killer app” for treating ovarian cancer. Several phase III trials have shown that addition of bevacizumab to standard chemotherapy with carboplatin and paclitaxel is highly efficacious for treating patients with advanced ovarian cancer [18,19]. A recent phase III trial (AURELIA) showed an increase in response rate and doubling of progression-free survival (6.7 vs. 3.4 months) in patients with ovarian cancer resistant to platinum-based chemotherapy who received single-agent chemotherapy plus bevacizumab compared with those who received chemotherapy alone; however, the effect of this regimen on overall survival (OS) has not been reported to date [21]. Bevacizumab has high therapeutic value; however, development of bevacizumab resistance is a major problem [22]. Emerging data suggest that bevacizumab resistance develops through two modes, namely, intrinsic and evasive (adaptive) modes. Development of bevacizumab resistance through the intrinsic mode depends on treatment history, tumor microenvironment, or genetic makeup while that through the evasive mode depends on the activation of alternative pro-angiogenic signaling pathways, including integrin-mediated pathways [23].

Considering the particular aspect of ovarian cancer dissemination, which is direct interaction between cancer cells and peritoneal organs, and the important contribution of angiogenesis, integrin-targeting therapies may be a potential new option for treating ovarian cancer. Integrins regulate diverse processes, including the adhesion, invasion, migration, proliferation, and survival of tumor cells as well as endothelial cells, fibroblasts, pericytes, bone marrow-derived cells, inflammatory cells, and platelets [9].

## 4. Biology of Integrins in Ovarian Cancer

Epithelial-mesenchymal transition (EMT) is an important process in EOC development through which epithelial cells acquire mesenchymal, fibroblast-like properties. A critical molecular feature of EMT is the downregulation of E-cadherin, a cell adhesion molecule expressed on the plasma membrane of most normal epithelial cells. Sawada et al. showed that E-cadherin downregulation upregulates the expression of fibronectin receptor α5β1-integrin, thus promoting the attachment of ovarian cancer cells to secondary metastasis sites such as the peritoneum and omentum [24]. They further showed that α5-integrin was overexpressed in 10 of 107 (9%) ovarian cancer tissues and that the median survival of patients with high α5-integrin levels was 26 months compared with 35 months of patients with low α5-integrin levels (*p* = 0.03). The important role of α5β1-integrin-fibronectin interaction in the adhesion of ovarian cancer cells to the mesothelium has been extensively analyzed. Casey et al. reported that α5β1-integrin and fibronectin mediated the formation of ovarian cancer spheroids and that treatment with anti-α5-integrin antibody inhibited the adhesion of these spheroids to the mesothelium [25]. Hu et al. measured Lewis y antigen and α5β1-integrin levels in EOCs and found that expression rates of Lewis y antigen and α5β1-integrin were significantly higher in drug-resistant ovarian cancers than in partially sensitive or sensitive ovarian cancers. They concluded that Lewis y antigen and α5-integrin overexpression was a strong risk factor of chemotherapeutic drug resistance in addition to surgical stage and residual tumor size in patients with ovarian carcinoma [26].

α6β2-Integrin is a cell adhesion molecule that binds to laminins in the extracellular matrix and nucleates the formation of hemidesmosomes. α6β4-Integrin is strongly expressed by many cancer cell types; however, few studies have reported its expression in ovarian cancer [27]. Villegas-Pineda et al. reported that 90% ovarian cancer tissues expressed α6β4-integrin [28]. The genomic profile of serous ovarian cancer is similar to that of basal-like breast cancer, with both the cancer subtypes showing frequent loss of *TP53*, *RB1*, and *BRCA1*, suggesting that β4-integrin plays an important role in both the cancer subtypes [29].

αvβ3-Integrin is preferentially expressed on developing rather than on mature vasculature and is the most important integrin for angiogenesis. Although the main ligand of αvβ3-integrin is vitronectin, it also interacts with fibronectin, fibrinogen, and thrombospondin [30]. αvβ3-Integrin is overexpressed in some cancers such as melanoma and breast cancer [31,32]. Landen et al. reported that αvβ3-integrin participated in the proliferation and invasion of ovarian cancer cells [33]. In ovarian cancer, the αv subunit has been found in cancer cells from effusion in 116 of 121 (96%) samples of patients [34]. In vitro migration of ovarian cancer cells depends on αvβ3-integrin. Gao et al. showed a close correlation between Lewis y antigen and αv-integrin expression and found that both Lewis y antigen and αv-integrin are independent drug resistance-related risk factors in patients with ovarian cancer [35]. In contrast, Kaur et al. reported that αvβ3-integrin-expressing ovarian cancer cells showed impaired invasion, protease expression, and colony formation and that patients with tumors expressing high β3-integrin levels showed significantly better prognoses than patients with tumors expressing low β3-integrin levels [36], indicating that this integrin subunit may exert detrimental effects on ovarian cancer progression.

The other important adhesion molecule which interact with ovarian cancer cells and the mesothelial cells is α4β1-integrin and vascular cell adhesion molecule-1 (VCAM-1) because VCAM-1 is preferentially expressed on the mesothelium of ovarian cancer cells [37]. α4β1-Integrin is commonly expressed on leukocytes, where it regulates leukocyte trafficking during infection and autoimmune diseases, including multiple sclerosis and Crohn’s disease [38]. Function-blocking antibodies against VCAM-1 and α4β1-integrin block the migration and metastasis of ovarian cancer cells in a xenografted model [39]. More recently, Scalici et al. reported that α4β1-integrin inhibition increased the response of ovarian cancer to carboplatin, suggesting that α4β1-integrin expression on ovarian cancer cells increases their resistance to platinum-based chemotherapy [38].

## 5. Clinical Trials of Integrin Inhibitors for Treating Ovarian Cancer

Great progress has been made toward targeting integrins in ovarian cancer. Several preclinical studies on various integrin antagonists have shown their effectiveness in blocking tumor progression. Integrin antagonists inhibit tumor progression by affecting both tumor cells and tumor-associated host cells, especially the angiogenic endothelium. Some of these integrin antagonists have progressed to clinical studies and have shown certain efficacies for cancer treatment.

**Figure 2:**
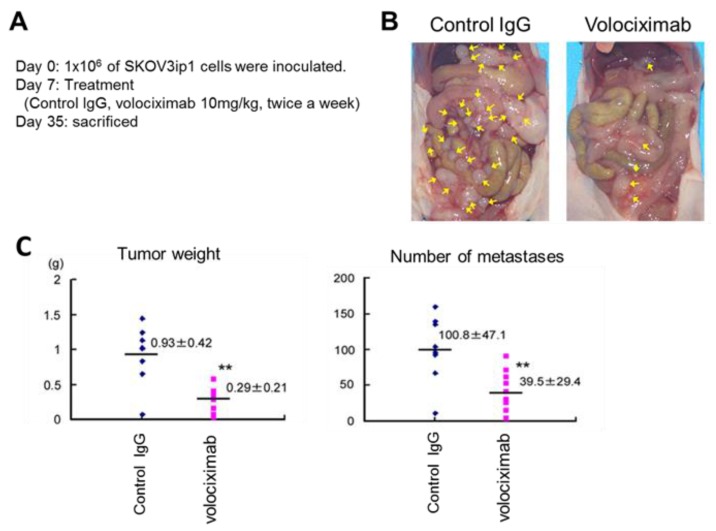
Blocking of α5β1-integrin (by volociximab) inhibits tumor metastasis in a xenografted model [42]. (**A**) Protocol: 1 × 10 ^6^ SKOV3ip1 cells were intraperitoneally injected into Balb-c female nude mice. The mice were randomized into two groups containing 10 mice each and were treated with 10 mg/kg volociximab or control IgG twice a week. On day 35 after the injection, the mice were sacrificed. (**B**) Representative images of each group. (**C**) Tumor weight (left) and number of tumor metastases are shown (** *p* < 0.01).

Volociximab, a high-affinity, chimeric antibody against human α5β1-integrin, inhibits endothelial cell survival and proliferation both in vitro and in vivo even after the adhesion of endothelial cells to the extracellular matrix through other integrins [40]. Thus, it inhibits tumor neoangiogenesis by blocking the interaction between α5β1-integrin and fibronectin and had been originally developed as an anti-angiogenic drug [41]. Mitra et al. showed that volociximab drastically reduced tumor burden in a xenografted model of ovarian cancer [42] (Figure 2), suggesting its potential for treating ovarian cancer. A phase I clinical biological correlative study reported the safety of long-term administration of volociximab in patients with advanced malignancies at doses up to 15 mg/kg per week [43]. A phase II, multicenter, single-arm, two-stage study on platinum-resistant, advanced epithelial ovarian or primary peritoneal cancer evaluated the efficacy, safety, and tolerability of weekly administration of single-agent volociximab at a dose of 15 mg/kg intravenously per week [44]. In this study, the efficacy of volociximab was assessed in 14 patients, of which one patient retained a stable disease for 8 weeks after the treatment, although the remaining 13 patients experienced disease progression during the treatment. Twelve (75%) patients developed study-related adverse events, with headache and fatigue being the most common (>20%) adverse events. Three patients developed possible study-related serious adverse events, namely, reversible posterior leukoencephalopathy syndrome, pulmonary embolism, and hyponatremia. Despite being well tolerated, volociximab showed insufficient activity in this study, partly because the patient population was very difficult to treat [44]. Zhang et al. reported that β1-integrin inhibition enhanced the effects of bevacizumab on the apoptosis, adhesion, and migration of ovarian cancer cells, suggesting that β1-integrin inhibition combined with bevacizumab treatment reduces the required dose of bevacizumab, thus potentially reducing drug-related morbidity [45]. α5β1-Integrin is essential for vascular development. Mice with homozygous α5-integrin knockdown show embryonic lethality because of severe vascular defects [46], suggesting that excess α5β1-integrin inhibition impairs the important physical activities of normal cells. To overcome this issue, Xie et al. suggested the specific inhibition of α5β1-integrin expression in ovarian cancer cells by using complementary replication-defective adenoviruses [47], speculating that the formation of ovarian cancer multi-cellular spheroids in ascites should be interfered. While several concerns such as hepatic tropism or innate and acquired immunity must be addressed for the clinical use of adenovirus vectors, recent adenovirus genomes have been engineered to overcome these problems [48]. Thus, the clinical usefulness of oncolytic adenovirus vectors would increase and those might be employed as integrin inhibitors in the near future.

Intetumumab (formerly called CNTO 95) is a human monoclonal antibody that recognizes all the members of αv-integrin family and has anti-angiogenic and antitumor properties. This pan anti-αv-integrin antibody binds to αv-integrins with high affinity and specificity, thus inhibiting cell adhesion, migration, proliferation, and invasion of both tumor and endothelial cells in vitro [49]. A phase I study of intetumumab showed its safety unlike that of other angiogenesis inhibitors; moreover, they found that intetumumab did not inhibit normal physiologic angiogenesis and showed antitumor activity [50]. Fluro-Deoxy Glucose-Positron Emission Tomography (FDG-PET) imaging showed complete response in one patient with ovarian carcinosarcoma that remained stable for 6 months after intetumumab treatment [50]. However, this drug failed to progress to phase II study; moreover, its efficacy against ovarian cancer has not been examined thus far. 

LM609, a mouse anti-human monoclonal antibody against αvβ3-integrin, showed considerable anti-angiogenic activity in preclinical trials [37]. Based on the results of these studies, etaracizumab (MEDI-522), a humanized version of LM609, was developed as one of the first integrin antagonists introduced in clinical trials. A phase I study involving 16 patients with advanced solid tumors reported the safety of etaracizumab at doses up to 6 mg/kg, with no evident immunogenicity [51]. Etaracizumab did not exert significant vascular effects such as hemorrhage or thromboembolic events; moreover, none of the patients receiving etaracizumab discontinued or delayed the treatment due to serious adverse events. Among the participants, five patients retained a stable disease for >6 months after the treatment [51]. A randomized phase II study of etaracizumab with or without dacarbazine in patients with stage IV metastatic melanoma reported a median OS of 12.6 months for patients receiving etaracizumab alone and of 9.4 months for patients receiving etaracizumab plus dacarbazine [52]. However, additional studies are needed to evaluate the efficacy of etaracizumab for treating solid tumors, including ovarian cancer.

Cilengitide is a selective inhibitor of αvβ3- and αvβ5-integrins [53]. A randomized phase II study of cilengitide combined with chemoradiation for treating newly diagnosed glioblastoma suggested that cilengitide alone or in combination with temozolomide chemoradiotherapy was well tolerated and showed potential antitumor activity [53,54]. A phase III, multicenter, open-label study investigated the efficacy of cilengitide in patients with newly diagnosed glioblastoma [55]. Results of this study showed that addition of cilengitide to temozolomide chemoradiotherapy did not improve the outcomes of patients with glioblastoma; therefore, the authors concluded that cilengitide cannot be further developed as an anticancer drug even though integrin-targeting therapy has the potential for treating glioblastoma [55].

## 6. Future Directions and Conclusions

Numerous integrin inhibitors have been evaluated clinically for a range of therapeutic indications. For instance, cilengitide alone has been studied in at least 35 clinical trials that have reported issues with its efficacy rather than its safety [56]. Until 2017, only αIIbβ3-integrin inhibitors (abciximab, eptifibatide and tirofiban) were approved for treating thrombosis because platelet αIIbβ3-integrin, also known as glycoprotein receptor-IIb/IIIa, is an important target for preventing clot formation [56]. Although various integrin inhibitors such as volociximab and etaracizumab have been assessed for treating solid tumors, including ovarian cancer, none of these inhibitors have shown sufficient efficacy for further clinical investigation. Agents targeting only a single integrin such as αvβ3- or α5β1-integrin have failed to show evident clinical benefits for treating metastatic cancers, indicating that more than one integrin-associated pathway is involved in cancer progression.

Recent studies have assessed RGD peptides as carriers for drug delivery. For instance, αvβ3-integrin is expressed on tumoral endothelial cells as well as on some solid tumor cells, including ovarian cancer cells. RGD peptides preferentially bind to αvβ3-integrin. Therefore, RGD peptides can be conjugated with drugs or radionuclides or can be grafted on the surface of nanoparticles (polymeric nanoparticles, liposomes, polymeric micelles, etc.) that encapsulate various agents such as anticancer drugs, peptides or proteins, nucleic acids, and radionuclides [57]. An in vivo preclinical study by Danhier et al. showed enhanced cellular uptake of paclitaxel-loaded RGD- conjugated nanoparticles in the tumor endothelium [58]. Meng et al. used liposomes as a nanocarriers to improve the solubility and specific targeting ability of paclitaxel toward the tumor vasculature in mice harboring A547 lung adenocarcinoma cells [59]. RGD peptides can be successfully integrated into lipid bilayers. RGD-integrated liposomes containing paclitaxel showed higher cellular uptake and resulted in lower tumor microvessel density than paclitaxel alone, indicating that integrin-based strategy can be used to enhance tumor-specific recognition of nanocarriers [59]. Because paclitaxel is the key drug for ovarian cancer treatment, such efforts may improve the chemoresensitivity of patients with refractory ovarian cancer in the near future.

Thus, although integrins play key roles in ovarian cancer metastasis and angiogenesis, none of the integrin inhibitors analyzed to date have shown favorable results, indicating that inhibition of a single integrin may not be sufficient to control ovarian cancer. However, RGD peptides addressed to α5β1-, αvβ3-, and αvβ6-integrin may be a promising drug delivery system, and integrin-targeting therapies may be a promising approach for further clinical investigation.
